# First-principles study of BX–SiS (X = As, P) van der Waals heterostructures for enhanced photocatalytic performance†

**DOI:** 10.1039/d3na00167a

**Published:** 2023-08-02

**Authors:** Sheraz Ahmad, H. U. Din, S. S. Ullah Sabir, B. Amin

**Affiliations:** a School of Materials Science and Engineering, Institute of New Energy Material Chemistry, Nankai University Tianjin 300350 P. R. China; b Computational Science Research Center, Korea Institute of Science and Technology (KIST) Seoul 02792 Republic of Korea 025264@kist.re.kr haleem.uddin@yahoo.com; c Department of Physics, Bacha Khan University Charsadda KP Pakistan; d Department of Physics, Hazara University Mansehra KP Pakistan; e Department of Physics, Abbottabad University of Science and Technology Abbottabad KP Pakistan

## Abstract

The vertical integration of two-dimensional (2D) materials through weak van der Waals (vdW) interactions is gaining tremendous attention for application in nanotechnology and photovoltaics. Here, we performed first-principles study of the electronic band structure, optical and photocatalytic properties of vertically stacked heterostructures based on boron pnictides BX (X = As, P) and SiS monolayers. Both heterobilayers possess a stable geometry and reveal type I band alignment with a direct band gap, indicating substantial transfer of charge across the junction of the same layer. Interestingly, a redshift is found in the visible light region of the optical absorption spectra of BX–SiS heterobilayers. The comparatively larger hole mobility (14 000 cm^2^ V^−1^ s^−1^) of BP–SiS preferably allows hole conduction in the zigzag-direction. More importantly, the excellent band edge values of the standard redox potential and smaller Gibbs free energy for the adsorption of hydrogen (Δ*G*_H*_) make them ideal for performing the hydrogen evolution reaction (HER) mechanism under solar irradiation. These findings offer exciting opportunities for developing next-generation devices based on BX–SiS heterobilayers for promising applications in nanoelectronics, optoelectronic devices and photocatalysts for water dissociation into hydrogen to produce renewable clean energy.

## Abstract 2

The vertical integration of two-dimensional (2D) materials through weak van der Waals (vdW) interactions is gaining tremendous attention for application in nanotechnology and photovoltaics.

## Graphical abstract

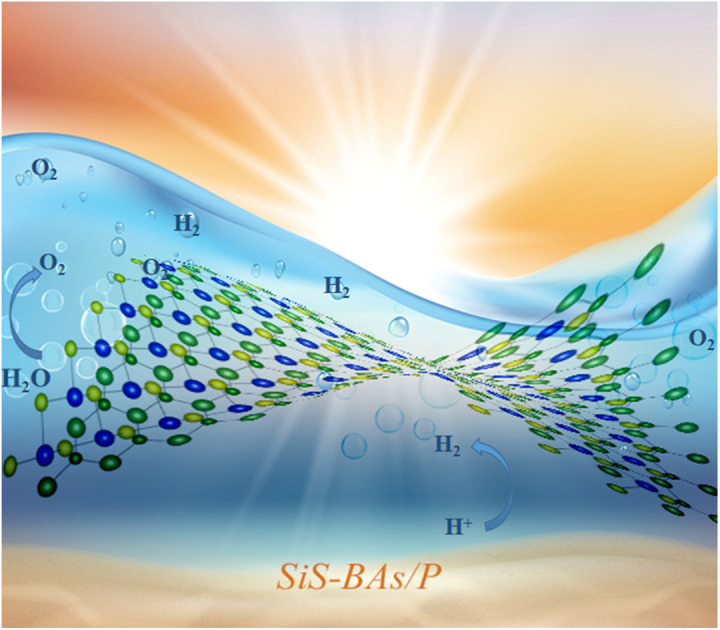

## Introduction

A technique that continues to improve for storing unlimited solar energy as chemical energy is solar energy conversion to hydrogen. Specifically, a complete conversion from solar energy to chemical energy, including light harvesting, energy transfer, and energy storage, is implemented *via* photocatalytic splitting of water into hydrogen.^1–3^ The majority of the photocatalysts, required for the water splitting, are semiconductors with the ability to separate charges and absorb light affecting the catalytic efficiency. Both the redox potential of the photogenerated carriers and the capacity to absorb light are controlled by the band structure of photocatalysts. Because of their narrow bandgaps, significant research has been conducted to create photocatalysts that have an extended light absorption zone, for instance, g-C_3_N_4_ (ref. 4) and Black P.^5^

After graphene was successfully exfoliated,^6–9^ research on the exploration of other 2D materials, including transition metal dichalcogenides (TMDs)^10–14^ and phosphorene,^15–18^ is largely under consideration. However, the disadvantages of these 2D materials are also becoming more evident and limit their application in electronic and optoelectronic devices.^19,20^ Among 2D materials, the SiS monolayer has gained tremendous attention owing to its strong anisotropic behavior for optical sensors, superior chemical and mechanical stability, high electron mobility, and promise as a candidate for solar cell applications.^21,22^ More recently, a novel class of two-dimensional materials, boron pnictides (BX, X = As, P) with a graphene-like planar honeycomb structure, were reported as promising candidates for innovative, multifunctional nanodevices in the field of nanotechnology.^23–28^

The formation of a van der Waals heterostructure enables tunable band alignment and offers a rational approach for enhanced optical absorption.^29,30^ For instance, a type-I heterostructure is constructed with the valence band maximum (VBM) and conduction band minimum (CBM) localized in a single constituent, which is crucial for usage in light-emitting or laser devices.^31^ When both the CBM and VBM are localized in different constituents it leads to type-II heterojunctions which are highly desirable for improving the photogenerated charge carriers' separation in photovoltaics.^32–34^ So far, heterostructures based on BAs/BP, such as MoSe_2_/BAs, have been analyzed and it has been reported that they possess a tunable bandgap and remarkable absorption of light with a conversion efficiency of 20%.^35^ According to Do *et al.* BP–ZnO and BAs–ZnO heterostructures show type I band alignment and good optical absorption, and promising as photocatalysts.^36^ It has been reported that type-II band alignment in GaN–SiS makes it suitable for photogenerated charge carrier separation and promising photocatalyst for water splitting.^37^ The BY–MX_2_ (BAs–WSe_2_ and BP–WS_2_) heterostructure exhibit a direct type-II (type-I) band alignment.^38^ Similar trends were observed in other vdW heterostructures including SiS/P (SiS/SiC),^39–41^ P–BSe and P/X_2_,^42^ and group III–VI and group IV–VI monolayer-based heterostructures.^43–45^ Despite these distinctive physical properties, the integration of BX and SiS-based vdW heterostructures remains unexplored for the practical design of new nano-based devices with exceptional functionalities.

Inspired by the excellent electronic properties and small lattice mismatch, the present work is devoted to studying the effect of vertically stacked BX and SiS monolayers by exploring the unprecedented optoelectronic and photodetection behavior. Herein, first principles calculations based on density functional theory are performed to confirm the structural stability, electronic band structure, transfer of charge, optical absorption, photocatalytic performance, and carrier mobility of BX–SiS vdW heterostructures. These structures have type II alignment of bands, doping of p-type in the BX-layer, good carrier mobility, redshift in the absorption spectrum, and improved photocatalytic properties. The findings may serve as a foundation for the practical application of BX–SiS in the design of next-generation photocatalysts, as well as photovoltaic and optoelectronic devices.

## Computational details

**Fig. 1:**
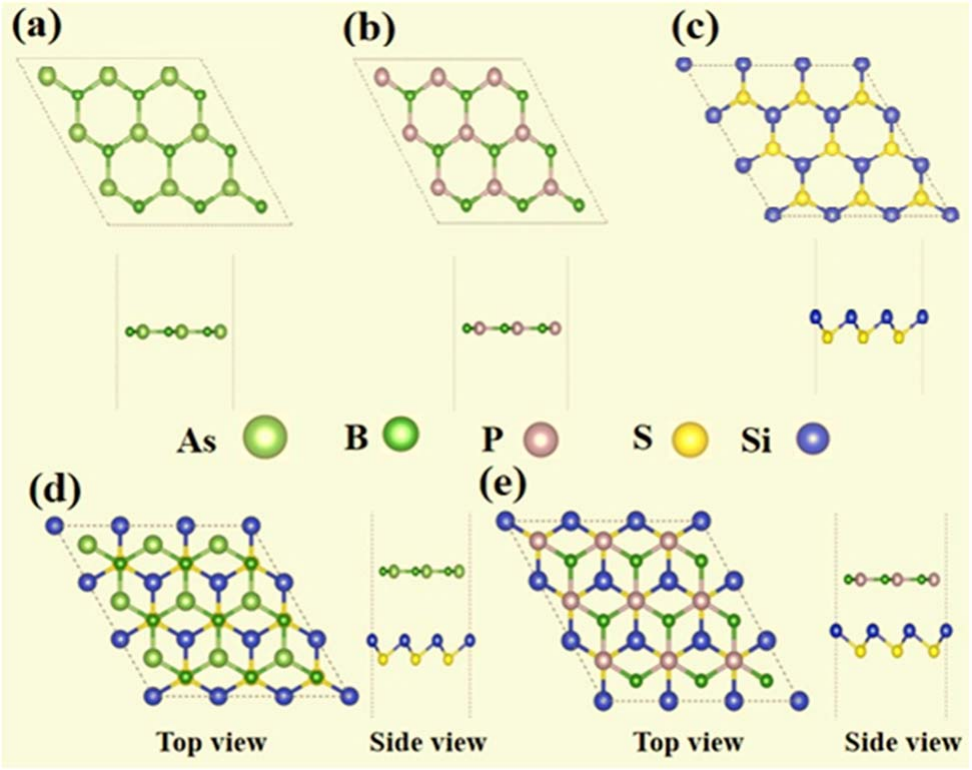
Top and side views of (a) BAs, (b) BP, and (c) SiS monolayers, and (d) BAs–SiS and (e) BP–SiS heterobilayers.

Electronic calculations were carried out by using a DFT-based simulation, the Vienna *Ab initio* Simulation Package (VASP).^46^ The projector augmented wave (PAW) method and generalized gradient approximation (GGA) were employed for the electron–ion interaction and exchange–correlation potential.^47,48^ The Brillouin zone^49^ was integrated with a 24 × 24 × 1 *k*-point grid for PBE^53^ and 12 × 12 × 1 *k*-mesh for HSE06 (ref. 50 and 51) calculation. The geometric relaxation of all systems were performed until the total energy and the residual forces on each atom reached 10^−5^ eV and 0.001 eV Å^−1^, respectively. A vacuum layer of 25 Å was introduced to prevent interaction with the nearby layer for making the vdW heterostructures. In addition, the Heyd–Scuseria–Ernzerhof (HSE) hybrid functional^60,61^ was employed to find the correct electronic band gap. To accurately describe the long-range weak vdW interaction we used Grimme's DFT-D3 method for the vdW interaction correction in the vdW heterostructure.^52,53^ The DFT-D3 method is widely accepted and incorporates corrections to the total energy, energy gradient, and frequencies of a molecule based solely on its molecular geometry. These corrections are added to the results obtained from conventional density-functional theory approximations and other mean-field approaches. One of the key advantages of DFT-D3 is its computational efficiency and applicability across the periodic table. To calculate the binding energy (*E*_b_) of the heterostructures we used the relation *E*_b_ = *E*_BX_–_SiS_ − *E*_SiS_ − *E*_BX_, where *E*_BX–SiS_, *E*_SiS,_ and *E*_BX_ represent the total energy of BX–SiS van der Waals heterostructures, SiS, and BX monolayer, respectively. The dynamical stability of the BX–SiS vdW heterostructure was investigated by employing density functional perturbation theory based Phonopy code to obtain the phonon vibration modes,^54,55^ wherein the atomic-displacement is regarded as a perturbed potential.^56^ A 4 × 4 × 1 supercell was used for calculating the correct force constant and phonon spectrum. Further, an *ab initio* molecular dynamics (AIMD) simulation approach is employed to check thermal stability.^57,58^ The Nose algorithm simulates every aspect of volume, lattice constant, and temperature. For the test sample, we chose a 4 × 4 × 1 supercell, and the total time was set to 10 000 fs with a time-step of 1 × 10^−15^ seconds (Fig. 1).

## Results and discussion

Electronic structures of boron pnictides BAs and BP, and SiS monolayers have been studied and revealed in the aforementioned work.^31–33^ Yuan S. *et al.* summarized a number of heterostructures made of 2D monolayers of group III–V and group IV–VI elements.^59–61^ The thermodynamic stability and small lattice-mismatch of BAs, BP, and SiS monolayers permit the computational designing of BX–SiS (X = As, P) van der Waals (vdW) heterostructures. The lattice mismatch is 2.6% and 2.4% for BAs–SiS and BP–SiS, respectively. Consequently, the structural configuration of boron pnictide BX (X = As, P) and SiS monolayers allows the design of six possible stacking patterns of the BX–SiS (X = As, P) vdW heterostructure (see details in Fig. S3(a)–(f) in the ESI†). Following the structural optimization of different possible configurations, the lattice constant, binding energy, and the interlayer separation (*d*_spacing_) between layers of BX, SiS monolayers and both BX–SiS heterobilayers are calculated. To ensure energetic stability, the binding energy (*E*_b_) of all six configurations of BX–SiS systems is calculated and presented in Table S1.† The stacking pattern a/b of the BAs–SiS/BP–SiS heterobilayer is energetically more feasible with reduced interlayer separation and greater binding energy, as shown in Tables 1 and S1.† These results are in good agreement with the available literature.^36–38,62,63^ Both monolayers and corresponding heterostructures have identical trends for dynamical stability, therefore, the phonon band spectrum is calculated for the systems under study, as displayed in Fig. S2† and 2. A careful inspection of the figure demonstrates a quadratic flexural phonon mode at the lowest acoustic branch at *Γ*-point (*z*-direction acoustic mode, ZA), which is known to be a common feature of 2D materials like silicene, germanene, and phosphorene.^64–66^ This can be attributed to structure-related reasons such as hybridization between the polarization of the TA/LA modes with the ZA mode, which usually occurs in nonplanar 2D materials. All of these can lead to a breakdown of the rotation symmetry and lead to a nonquadratic ZA branch. However, the presence of the U-shaped region with an imaginary frequency in the phonon spectra does not correspond to the instability of 2D materials. Furthermore, some low-frequency modes are located at the *Γ*-point responsible for van der Waals (vdW) coupling between the stacked monolayers and indicate the construction of a heterostructure. Additionally, *ab initio* molecular dynamics simulations (AIMDS) are essentially important to ensure the thermal stability of the most feasible stacking structure at room temperature. The energy fluctuation *versus* time of the BX, SiS monolayers, and BX–SiS vdW heterostructures under study is calculated, as displayed in Fig. S1(a)–(c)† and 3(a) and (b). The final structures have no broken bond at 10 000 fs, indicating the feasible fabrication of BX–SiS vdW heterostructures at 300 K.

**Table 1:** Lattice constant (*a* in Å), bond length (in Å), interlayer distance (*d*_spacing_ in Å), band gap (*E*_PBE_ and *E*_HSE06_ in eV), work-function (*ϕ* in eV), charge transfer (CT in eV), potential-drop (Δ*V*), and valence and conduction band edges (*E*_VB_ and *E*_CB_ in eV) of the pristine monolayers and corresponding heterostructures

Parameters	BAs	BP	SiS	BAs–SiS	BP–SiS
*a* = *b* (Å)	3.33	3.25	3.33	3.33	3.25
Bond length (Å)	1.92	1.859	2.38	1.92–2.33	1.87–2.35
*d*_spacing_ (Å)	—	—	—	3.5	3.3
PBE-*E*_g_ (eV)	0.71	0.93	2.2	0.8	0.79
HSE06-*E*_g_ (eV)	1.39	1.62	3.01	1.4	1.42
*ϕ* (eV)	—	—	—	4.72	5.01
CT	—	—	—	0.017	0.007
Δ*V* (eV)	—	—	—	0.1	3.68
*E*_VB_ (eV)	0.965	1.216	2.44	1.3	1.37
*E*_CB_ (eV)	−0.425	−0.174	−0.54	−0.11	−0.05

**Fig. 2:**
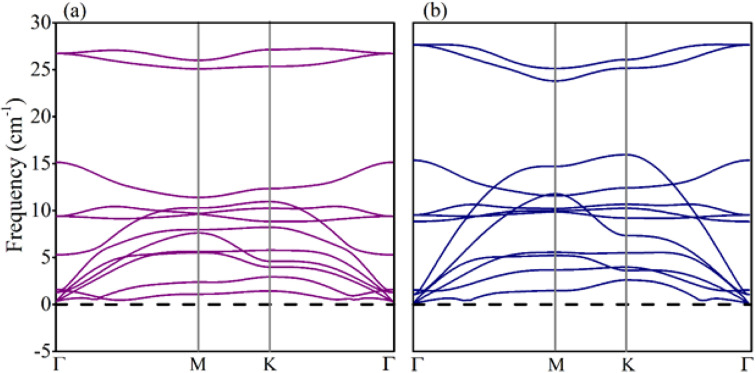
(a) Phonon band structures of (a) BAs–SiS and (b) BP–SiS vdW heterostructures.

**Fig. 3:**
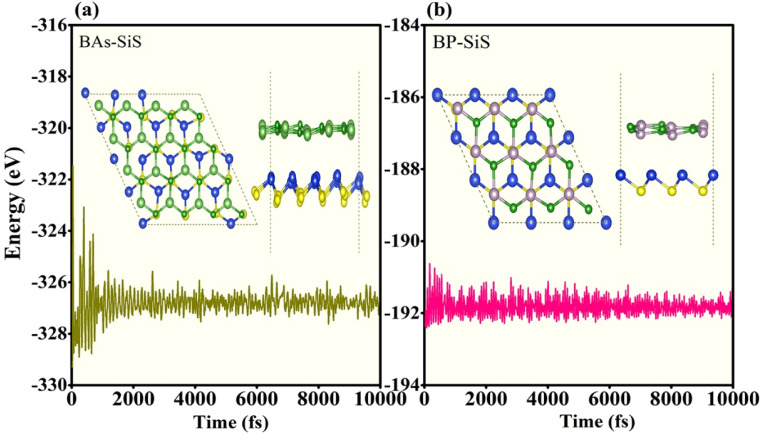
The AIMD calculation of energy fluctuations *versus* time (fs) of (a) BAs–SiS and (b) BP–SiS heterobilayers, where the inset represents the final structure at 300 K.

**Fig. 4:**
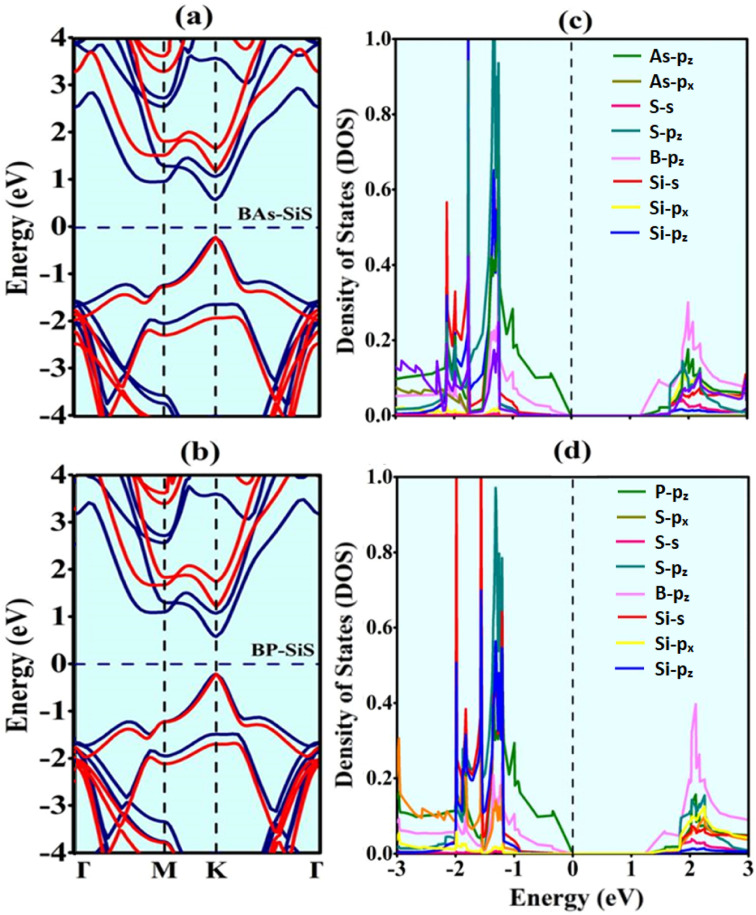
(a and b) Electronic band-structures and (c and d) partial density of states (PDOS) of BX–SiS vdW heterostructures. In (a and b), the PBE- and HSE06-functionals are represented by blue and red solid lines, respectively.

To analyze electronic and optoelectronic applications, exploration of the electronic characteristics of vdW heterostructures is needed. Using Generalized-Gradient-Approximation-PBE and HSE06 functionals, the electronic band structures of BAs, BP, SiS, and corresponding BX–SiS heterobilayers are calculated, as shown in Fig. S3(a)–(c)† and 4(a) and (b). As shown in Fig. 4(a) and (b), it is obvious that all heterostructures have a direct bandgap with VBM/CBM located at the *K* point in a Brillouin zone. Table 1 clearly shows that using the HSE06 functional, bandgap values at *K* points are greater than those obtained using the PBE-functional. A similar trend has been demonstrated in group III–V and group IV–VI 2D materials,^59–61^ BX–ZnO,^36^ GaN–SiS,^37^ and BY–MX_2_ (BAs–WSe_2_ and BP–WS_2_)^38^ have all shown similar trends. Additionally, the partial density of states (PDOS) of the pristine monolayers and vdW heterostructures under study, as shown in Fig. S4(d)–(f)† and 4(c) and (d), is used to calculate the contribution of each state near the Fermi level (*E*_F_). The VBM is predominantly contributed by the As/P-p_*z*_ orbital and CBM is largely attributed to the B-p_*z*_ orbital of the BX monolayer in BAs–SiS and BP–SiS hetero-bilayers. It is obvious that both the VBM and CBM are contributed by the same monolayers demonstrating straddling alignment of the band. This behavior is essential for application in light-emitting and laser devices as found theoretically in BAs/BP^67^ and SiS/P/SiC van der Waals hetero-bilayers.^68^

**Fig. 5:**
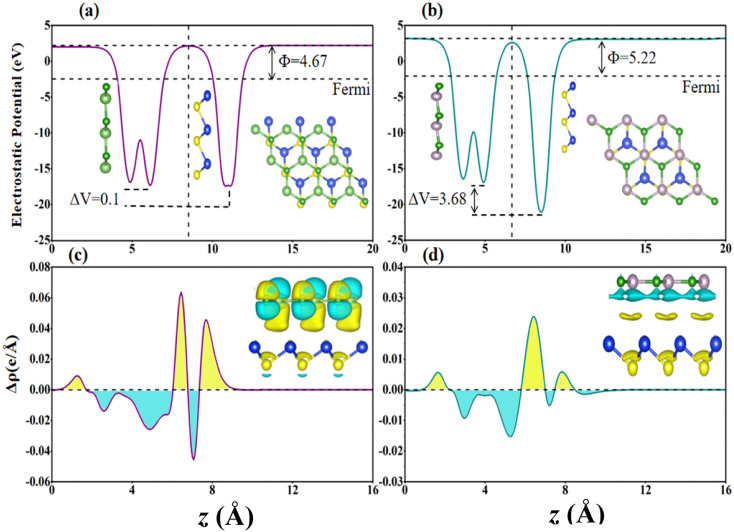
Charge density difference with yellow and cyan representing charge accumulation and depletion, and 0.0025*e* Bohr^−3^ being the iso-surface value for (a) BAs–SiS and (b) BP–SiS vdW heterostructure systems. Planar-averaged charge density difference of (c) BAs–SiS and (d) BP–SiS vdW heterostructure systems.

Furthermore, the charge density difference (Δ*ρ* = *ρ*_BX–SiS_ − *ρ*_BX_ − *ρ*_SiS_) and planar averaged charge density difference (Δ*ρ*_avg_(*z*)) in the *z*-direction are calculated to obtain a detailed understanding of the charge transfer and binding mechanism between the layers of (a) BAs–SiS and (b) BP–SiS vdW heterostructure, shown in Fig. 5(c) and (d), respectively, where *ρ*_BX–SiS_, *ρ*_BX_, and *ρ*_SiS_ represent the charge densities of the BX–SiS hetero-bilayer, BX, and SiS monolayer, respectively. Fig. 5(c) and (d) show the accumulation and depletion of charge as represented by the positive Δ*ρ*_avg_(*z*) yellow and negative Δ*ρ*_avg_(*z*) cyan regions, respectively. Reallocation of charge is mostly detected between P/As atoms and S because of the difference in electronegativity. Additionally, it is evident that electrons migrate from the BX to the SiS layer. This causes p-type doping in the BX layer and n-type doping in the SiS layer, causing an intrinsic electric field and serving as a driving push for the separation of photogenerated electron–hole pairs in different component monolayers. Additionally, the quantitative charge transfer is confirmed by the Bader charge analysis, which also reveals that the SiS layer acquires electrons while the BX layer loses electrons, as shown in Table 1. This negligible transfer in charge shows how little the BX and SiS monolayers interact. Other materials have demonstrated a similar tendency of weak interaction.^36,38^

**Fig. 6:**
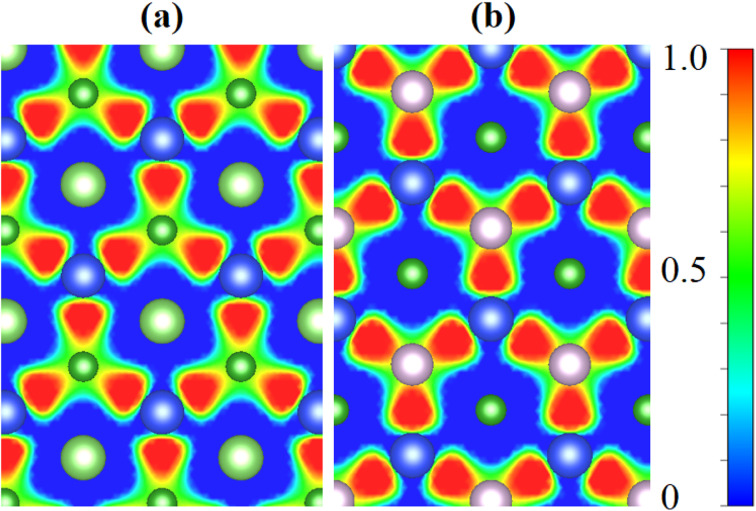
Electron localization function of (a) BAs–SiS and (b) BP–SiS vdW heterostructure systems.

Further, we explored the underlying mechanism of charge redistribution at the interface by computing the junction's planar averaged electrostatic potential (EP) along the *z*-axis for both vdW heterostructures, as displayed in Fig. 5(a) and (b). In Table 1, one can see the computed potential drop and work function across the interfaces of the BAs–SiS and BP–SiS vdW heterostructures. We found that the Fermi energy of the BX monolayer is lower than that of the SiS monolayer when the work functions of monolayers are compared. Typically, electrons display a tendency of being driven out of the BX and SiS monolayers in BX–SiS as long as the two Fermi energies are not equal. The electron transport from the BX layer to the SiS layer is clearly shown in Fig. 5(a) and (b) by the deeper potential in the BX layer than in SiS. Furthermore, in BX–SiS hetero-bilayers, the higher potential drop (Δ*V*) substantially reduces the rate of recombination of photogenerated carriers (holes and electrons) by creating a powerful electrostatic field at the interface. This increases driving and separates charge carriers, improving the efficiency of the power conversion process.^69–72^ The electron localization functions (ELF) were carried out in order to better understand the binding characteristics between two monolayers and gain a deeper understanding of the heterostructure behavior. ELF maps projected on (110) planes for the stoichiometry of BAs–SiS and BP–SiS are shown in Fig. 6. More specifically, the ELF value ranges from 0 to 1 and represents the likelihood of discovering an electron with the same spin as the reference electron. The enhanced localization of the electrons is correlated with the higher value of ELF. The electrons are totally localized when ELF = 1. Values close to zero indicate regions with low electron densities, and ELF = 0.5 is attributed to a homogeneous electron gas with metallic bond characteristics.

**Fig. 7:**
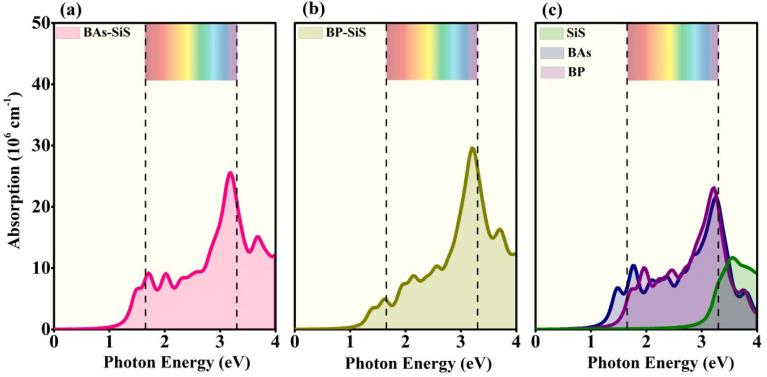
Optical-absorption spectrum of (a) BAs–SiS, (b) BP–SiS vdW heterostructures, and (c) SiS, BP, and BAs monolayers.

The optical characteristics of a material are important for understanding the light absorption in the material to promote effective photocurrent conversion. The absorption spectra of BX–SiS heterostructures are provided in Fig. 7. Because of the smaller bandgap in comparison to the corresponding BX and SiS monolayers, the redshift in the absorption spectra for BX–SiS vdW heterostructures increases systematically in the visible region of the electromagnetic spectrum. This redshift results in the splitting of excitons by an internal electric field across the region of the interface. The widening of absorption spectra in visible regions or lower energy indicates that BAs–SiS and BP–SiS vdW heterobilayers are suitable candidates for applications in photovoltaics and photocatalysis.

**Fig. 8:**
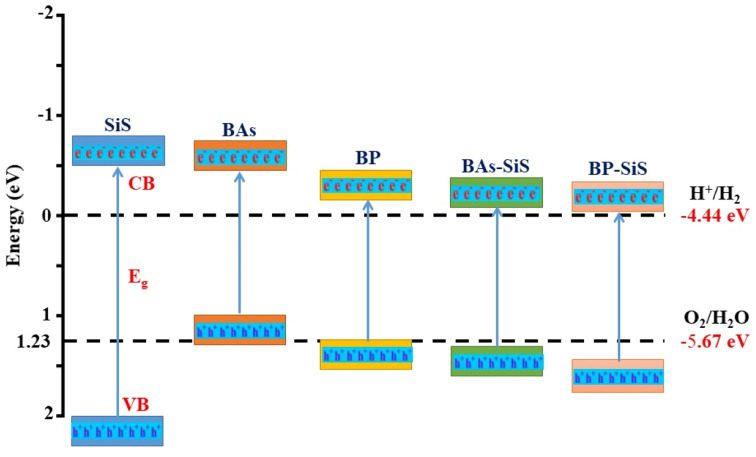
Schematic representation of the valence and conduction band edges of SiS, BP, and BAs monolayers and BAs–SiS and BP–SiS vdW heterostructures for photocatalytic splitting of water.

The photocatalytic characteristics of recently developed semiconductors that are capable of photocatalytic water splitting are then investigated. Preferably, for efficiently absorbing solar light, the value of the band gap for a semiconductor should be equal to or greater than 1.23 eV.^59–61,71^ Analysis of the proper positioning of the valence and conduction band (*E*_VB_ and *E*_CB_) edges in relation to the photo-excited charge carriers leads to the determination of BX–SiS heterostructures' capability for photocatalytic activity. Using the HSE06 functional, the positions of band edges for BX–SiS vdW heterostructures are determined, as shown in Fig. 8 and listed in Table 1. Using the following empirical equations,^73,74^ one can compute the standard reduction and oxidation potentials for water splitting:*E*_red_ = −4.44 eV + pH × 0.059 eV

And*E*_oxd_ = −5.67 eV + pH × 0.059 eV

A perfect photocatalyst for splitting water needs *E*_CB_ higher than −4.44 eV and *E*_VB_ lower than −5.67 eV at pH = 0, where −4.44 and −5.67 eV are the redox potentials. The relative positions of the *E*_VB_ and *E*_CB_ for understudied hetero-bilayers are found to be higher than the standard reduction potentials for H^+^/H_2_ and lower than the standard oxidation potential for O_2_/H_2_O, respectively (see Fig. 8). To be more precise, both BAs–SiS and BP–SiS vdW heterostructures have the ability to split water using solar energy without the use of external voltage. Similar trends have also been predicted theoretically in the published studies.^44,45,59–61,75,76^ These findings revealed that BAs–SiS and BP–SiS vdW heterostructures are promising candidates for applications in photovoltaics and photocatalytic water splitting.

**Fig. 9:**
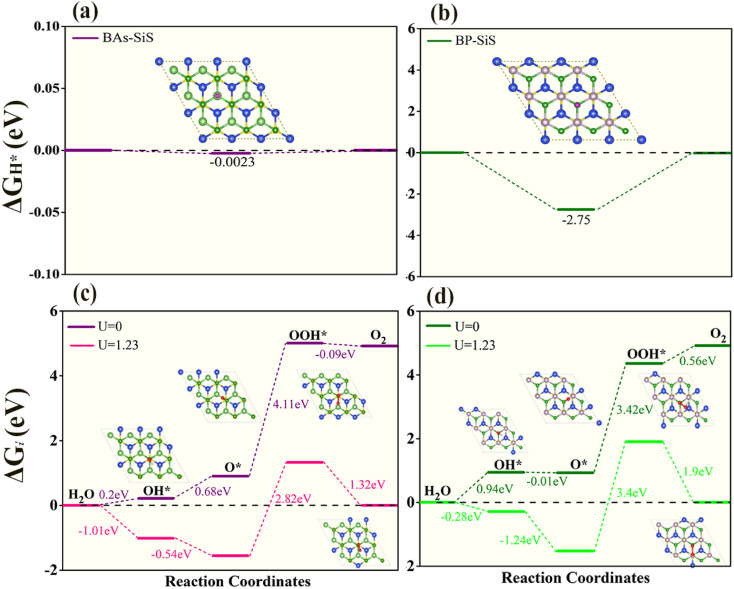
Free-energy diagrams for the HER and OER of (a and c) BAs–SiS and (b and d) BP–SiS vdW heterobilayers. Insets show the top view of the adsorbed models.

Finally, the HER performance of photocatalytic water splitting of BAs–SiS and BP–SiS vdW heterostructures was investigated. The hydrogen evolution reaction (HER) is a chemical reaction useful to produce H_2_.^77^ According to the Volmer–Heyrovsky–Tafel reaction mechanism, initially, the hydrogen proton and an electron are combined, the H atom is adsorbed on the electrode in the intermediate state, and in the final stage, a hydrogen molecule is produced. These stages are explained by the reactions given below:

i H^+^ + e^−^ → H*

ii H* + H^+^ + e^−^ → H_2_

iii H* + H* → H_2_

where H* represents adsorbed hydrogen on an adsorption site. The Gibbs free energy, Δ*G*_H*_, between the intermediate and final stages, has been shown to be a helpful descriptor for the HER. The activity of the HER is inversely related to the absolute value of the Gibbs free energy for the adsorption of hydrogen (Δ*G*_H*_). A catalyst is considered to be suitable for the HER if Δ*G*_H*_ = 0, that is Δ*G*_H*_ is nearly equal to the thermoneutral value. Here, we calculated Δ*G*_H*_ for one distinct adsorption site referred to as the B site on both SiS–BP and SiS–BAs vdW heterostructures as shown in Fig. 9(a) and (b). It is evident from Fig. 9 that Δ*G*_H*_ is closer to zero on the As site for BAs–SiS compared to the BP–SiS heterostructure, suggesting that the BAs–SiS heterostructure exhibits better HER performance. The HER performance of BP–SiS and BAs–SiS heterostructures has been found to be comparable to that of the most active catalyst for HER activity,^78^ as described by our calculated Gibbs free energy of adsorption. Furthermore, the oxygen evolution reaction (OER) is a four-electron water oxidation process, and the probable reaction mechanism steps are as follows:^79,80^

iv H_2_O → O_2_ + (4H^+^ + 4e^−^)

v H_2_O + * → OH* + (H^+^ + e^−^)

vi OH* → O* + (H^+^ + e^−^)

vii O* + H_2_O → OOH* + (H^+^ + e^−^)

viii OOH* → O_2_ + (H^+^ + e^−^)

where the asterisk (*) sign represents the BX–SiS surface and OH*, O*, and OOH* denote an adsorbed oxygenated species, respectively. Finally, oxygen is released. The change in Gibbs free energy (Δ*G*_1_ = Δ*G*_OH*_, Δ*G*_2_ = Δ*G*_O*_ − Δ*G*_OH*_, Δ*G*_3_ = Δ*G*_OOH*_ − Δ*G*_O*_, and Δ*G*_4_ = 4.92 − Δ*G*_OOH*_) for the OER process (from reaction steps v–viii) is separately calculated (see Fig. 9(c) and (d)). Without irradiation (at *U* = 0 eV), the reaction steps corresponding to the formation of OH*, O*, and OOH* are exothermic while the last step for the formation of O_2_ is endothermic for BAs–SiS systems. In contrast, the reaction steps for the formation of OH*, OOH*, and O_2_ are exothermic (except for O*) for the BP–SiS system. However, the photogenerated holes provide a driving force under solar irradiation (*U* = 1.23 eV), decreasing the Δ*G* value for OH* and O* reaction steps. The third step to form OOH* goes uphill with a higher value of Δ*G* (see Fig. 9(c) and (d)). This step is unstable and OOH* donates a proton to form an O_2_ molecule. The maximum uphill free energy at *U* = 1.23 eV corresponds to the thermodynamic rate-determining step (RDS). Obviously, the RDS of BP–SiS exhibits a larger free energy (3.4 eV) than BAs–SiS (2.82 eV), suggesting that the OER catalytic activity of BAs–SiS is better than that of BP–SiS.

The electronic performance of 2D BX–SiS vdW heterostructures is significantly influenced by carrier mobility, including electron mobility and hole mobility. So next, we estimated the carrier mobility (*μ*) using Bardeen and Shockley's deformation theory.^81^ The carrier mobility in 2D materials can be described as:

ix *μ* = 2*e*ℏ^3^*C*_2D_/3*k*_B_*T*(m*)^2^*E*_1_^2^

Here, *C*_2D_ is the 2D elastic modulus, *T* is the temperature, m* is the effective mass, and *E*_1_ is the deformation potential constant, which increases in direct proportion to the strain-induced band edge shift. The *E*_1_ is determined through the linear fitting of the VBM (CBM)–strain relation. Similarly, *C*_2D_ is computed by quadratically fitting the total energy *vs.* strain. The carrier mobility is calculated by using eqn (ix) for the available *C*_2D_, *E*_1_, and m* values. The corresponding data for each monolayer (BAs, BP, and SiS) and their two vdW heterostructures along the zigzag (*Z*) and armchair (arm) directions for electrons and holes are given in Table 2.

**Table 2:** Calculated two-dimensional elastic modulus (*C*_2D_), effective mass (m* along the *x* and *y* directions), deformation potential constant (*E*_1_), and mobility of charge carriers (*μ*) in the zigzag (*z*) and armchair (arm) directions of the (a) BAs, (b) BP, and (c) SiS monolayers and (d) BAs–SiS and (e) BP–SiS vdW heterostructures at 300 K

Materials	Charge	*C*_2D_	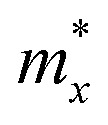	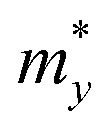	*E*_1_	*μ* (10^3^ cm^2^ V^−1^ S^−1^)
SiS	*e*_*z*_	39.39	0.45	1.514	1.714	2.837
	*e*_arm_	7.14	0.46	2.514	2.5	0.0328
	*h*_*z*_	39.39	4.82	3.514	1.92	0.0177
	*h*_arm_	7.14	2.84	4.514	0.98	0.0150
BAs	*e*_*z*_	168.08	0.16	5.514	2.73	0.6360
	*e*_arm_	27.11	0.23	6.514	5.53	0.0143
	*h*_*z*_	168.08	0.16	7.514	1.46	1.5576
	*h*_arm_	27.11	0.21	8.514	2.92	0.0416
BP	*e*_*z*_	140.50	0.20	9.514	2.30	0.3232
	*e*_arm_	26.66	0.31	10.514	4.67	0.0086
	*h*_*z*_	140.50	0.19	11.514	0.928	1.700
	*h*_arm_	26.66	0.27	12.514	1.88	0.0506
BAs–SiS	*e*_*z*_	215.49	0.21	13.514	2.72	0.2315
	*e*_arm_	32.98	0.26	14.514	5.17	0.0073
	*h*_*z*_	215.49	0.19	15.514	2.05	0.3892
	*h*_arm_	32.98	0.26	16.514	4.05	0.0104
BP–SiS	*e*_*z*_	201.94	0.20	17.514	2.47	0.2102
	*e*_arm_	52.2	0.25	18.514	4.90	0.0104
	*h*_*z*_	201.94	0.20	19.514	0.28	14.6097
	*h*_arm_	52.2	0.26	20.514	0.72	0.41709

The 2D modulus for BAs–SiS (BP–SiS) along the zigzag (zig) direction is about seven (four) times greater than that along the armchair (arm) direction. The effective mass exhibits an anisotropic behavior: for BAs–SiS (BP–SiS) in a zigzag direction, the hole-effective mass is smaller than (equal to) that for an electron, while, for the armchair direction, the effective mass of the electron is equal to (smaller than) that of the hole. Highly anisotropic carrier mobilities are predicted for the BX, SiS, and corresponding vdW heterostructures. The computed electron mobility value for BAs–SiS (BP–SiS) in the zigzag direction is 0.231 × 10^3^ cm^2^ V^−1^ s^−1^ (0.21 × 10^3^ cm^2^ V^−1^ s^−1^), which is 33 (21) times greater when compared with the mobility in the armchair direction, 0.007 × 10^3^ cm^2^ V^−1^ s^−1^ (0.01 × 10^3^ cm^2^ V^−1^ s^−1^). In contrast, the mobility of holes in the zigzag direction is 0.389 × 10^3^ cm^2^ V^−1^ s^−1^ (14.609 × 10^3^ cm^2^ V^−1^ s^−1^), nearly 4 (35) times greater in comparison to that in the armchair direction, 0.01 × 10^3^ cm^2^ V^−1^ s^−1^ (0.417 × 10^3^ cm^2^ V^−1^ s^−1^). Notably, the carrier mobilities, calculated for the vdW heterostructures under study, are higher than those of the monolayer of MoS_2_ (60–200 cm^2^ V^−1^ s^−1^).^81^ Particularly, BP–SiS heterobilayers show that the mobility of holes in the zigzag direction is nearly 70 times larger than electron mobility, enhancing the potential for hole conduction in the zigzag direction. Higher carrier mobility and a smaller effective mass^82^ result in greater transport of charge carriers, which is desirable for functional electronic and optoelectronic devices. Consequently, the calculated carrier mobility and effective mass of electrons and holes show that the monolayers of BX, SiS, and their BAs–SiS and BP–SiS heterostructures are promising for applications in high-performance electronic devices. A significant difference in the mobility of charge carriers is exploited for the separation of electrons and holes.

## Conclusion

In summary, we employed density functional theory-based first principles calculations to explore the structural, electronic, optical, and photocatalytic characteristics of BX (X = As, P) and SiS monolayers and their vdW heterostructures BAs–SiS and BP–SiS. Both vdW heterostructures exhibited energetic, dynamic, and thermal stability. Our findings show that owing to the weak vdW interactions occurring in the heterostructures, the electronic properties of BX–SiS vdW heterostructures seem to be the combinations of constituent layers. Interestingly, BX–SiS heterostructures are direct band gap semiconductors with type I band alignment. A built-in electric field across the junction leads to charge transfer from the BX to the SiS layer, responsible for p-type doping in the BX layer. This reduces the rate of photogenerated charge recombination. Red-shift and widening of the absorption spectra in the range of visible light suggest that BX–SiS heterostructures are suitable candidates for application in solar light conversion. Both heterostructures are capable of the reduction of water into H^+^/H_2_ and oxidation into O_2_/H_2_O. The in-plane carrier mobilities of the two-dimensional BX–SiS heterostructures are vastly anisotropic. Particularly, in the BP–SiS vdW heterostructure in the zigzag direction, the hole mobility (14 000 cm^2^ V^−1^ s^−1^) is nearly 70 times larger than the mobility of electrons, enhancing the potential for hole conduction in the *z*-direction. These promising heterostructures offer exciting opportunities for developing next-generation optoelectronic devices and photocatalysts for water dissociation into hydrogen to produce renewable clean energy.

## Conflicts of interest

There are no conflicts to declare.

## Supplementary Material

NA-005-D3NA00167A-s001
