# Supplementary material for: First-principles study of BX–SiS (X = As, P) van der Waals heterostructures for enhanced photocatalytic performance
Source: Nanoscale Adv. 2023 Aug 2;5(17):4598–608. doi: 10.1039/d3na00167a (PMC10448330; doi:10.1039/d3na00167a)
Supplement: NA-005-D3NA00167A-s001 [file NA-005-D3NA00167A-s001.pdf]

Electronic Supplementary Material (ESI) for Nanoscale Advances.
This journal is © The Royal Society of Chemistry 2023

First-principles study of BX-SiS (X=As, P) van der Waals heterostructures for enhanced photocatalytic performance

Sheraz Ahmad [1], H.U. Din [2,3,*], S.S.Ullah Sabir [4], B. Amin [5]

[1] School of Materials Science and Engineering, Institute of New Energy Material Chemistry, Nankai University, Tianjin 300350, P. R. China
[2] Computational Science Research Center, Korea Institute of Science and Technology (KIST), Seoul 02792, Republic of Korea.
[3] Department of Physics, Bacha Khan University, Charsadda, KP, Pakistan.
[4] Department of Physics, Hazara University, Mansehra, KP, Pakistan
[5] Department of Physics, Abbottabad University of Science and Technology, Abbottabad, KP, Pakistan.

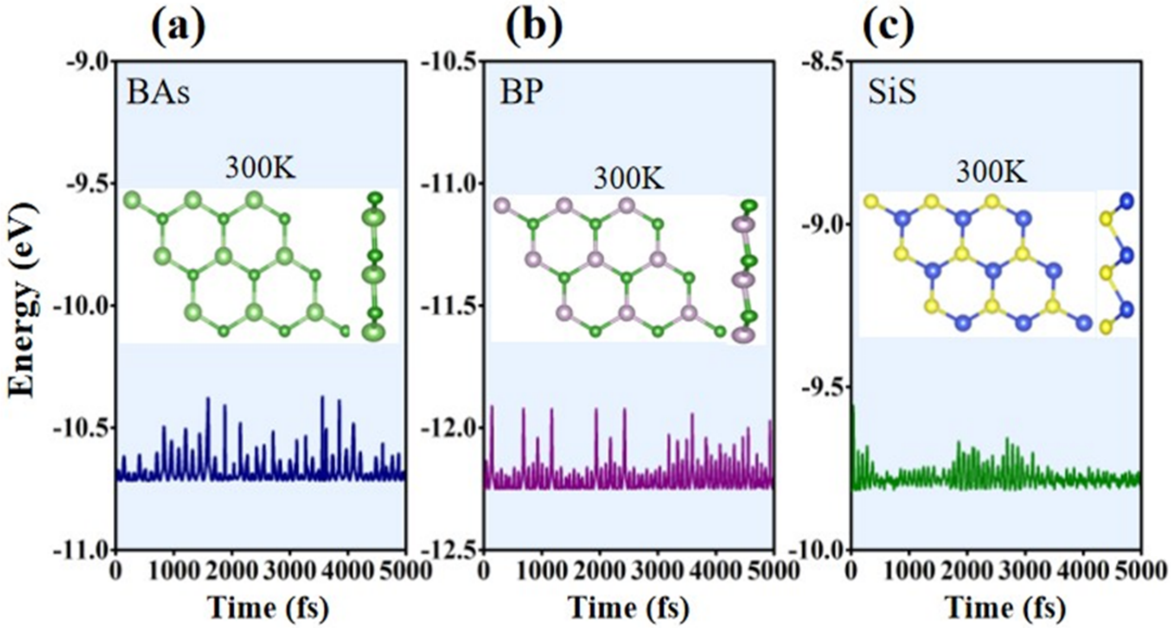

Fig. S1. The energy versus time (fs) plots of (a) BAs (b) BP (c) SiS monolayers with insets representing the final structure of pristine monolayers at 300K.

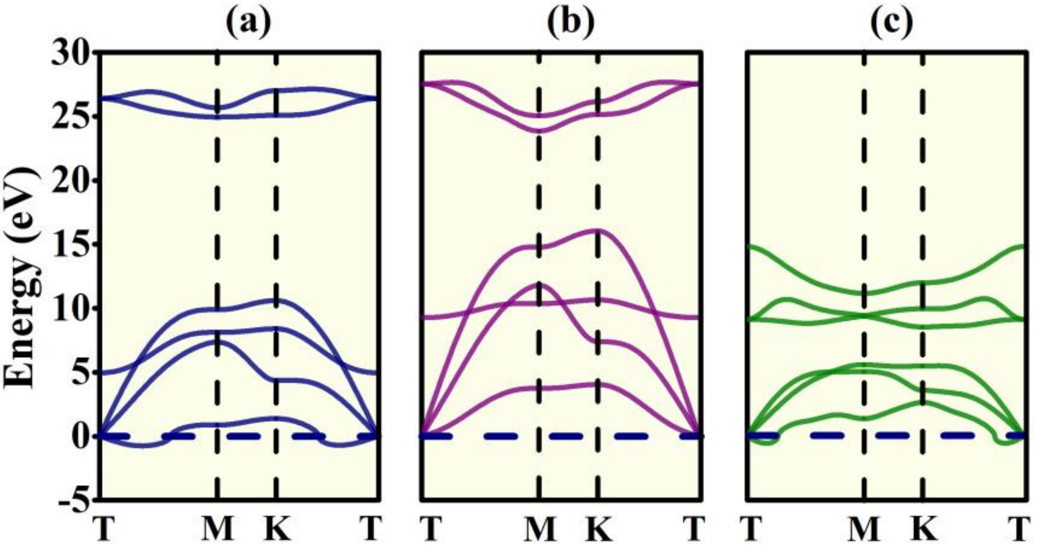

Fig. S2. (a) Phonon band structures of (a) BAs (b) BP (c) SiS monolayers.

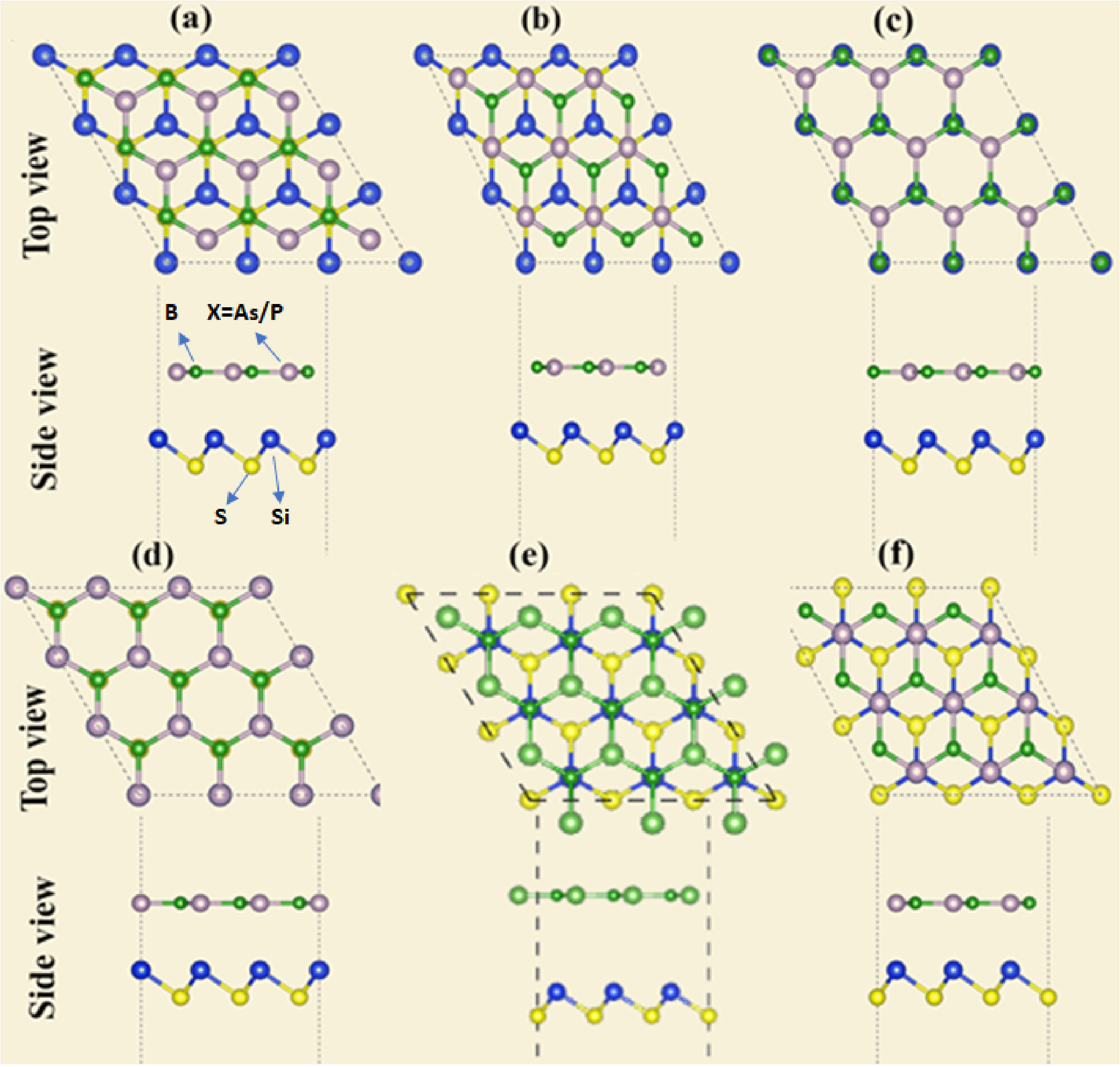

Figure S3. Stacking configurations of BX-SiS heterostructures, (a/b). B(X)-atom is placed on top of S-atom and X(B)-atom is located on hollow site. (c/d). X(B)-atom is positioned on top of S(Si)-atom and B(X)-atom is localized on Si(S)-atom. (e/f). B(X)-atom is lying on Si-atom and X(B)-atom occupies the hollow center.

Table S1. The binding energy (E_b in eV/Å^2) of six stacking configurations of both BX-SiS (X=As, P) heterostructures.

**Configurations**	**BAs-SiS**	**BP-SiS**
	**E_b (eV/Å^2)**	**E_b (eV/Å^2)**
a	-0.04925	-0.04504
b	-0.04915	-0.04547
c	-0.04540	-0.04285
d	-0.04332	-0.04077
e	-0.04540	-0.04285
f	-0.04223	-0.03968

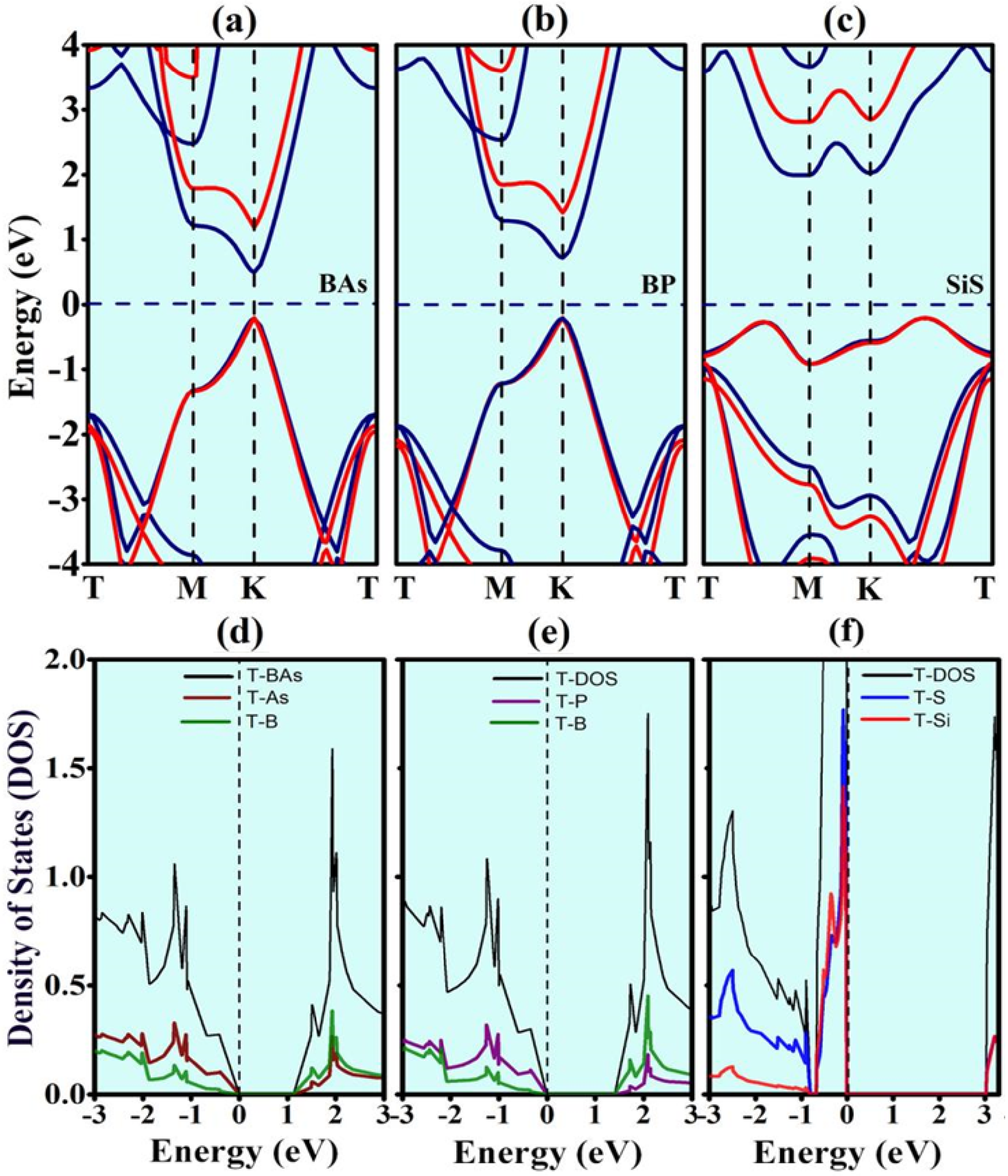

Figure S4. Electronic band structures (a-c) and partial density of states (PDOS) (d-f) of pristine BAs, BP, and SiS monolayers, respectively.
